# The complete chloroplast genome of *japonica* type weedy rice (*Oryza sativa* f. *spontanea*)

**DOI:** 10.1080/23802359.2022.2106160

**Published:** 2022-08-01

**Authors:** Yiyu Hu, Chenfeng Dong, Chuyu Ye, Longjiang Fan, Wei Tang

**Affiliations:** a Zhongyuan Institute, Zhejiang University, Zhengzhou, China; b Institute of Crop Sciences, College of Agriculture and Biotechnology, Zhejiang University, Hangzhou, China; c State Key Laboratory of Rice Biology, China National Rice Research Institute, Hangzhou, China

**Keywords:** *Oryza sativa* f. *spontanea*, chloroplast genome, weedy rice

## Abstract

As a noxious weed, weedy rice (*Oryza sativa* f. *spontanea* Roshev. 1931) has threatened global food security and sustainable crop production. On the other hand, weedy rice has a strong tolerance for abiotic stresses and the potential to provide rich resources for rice genetic improvement. Thus, for a more comprehensive understanding of its speciation, we sequenced and assembled the first complete chloroplast genome of *Oryza sativa* f. *spontanea* (*japonica* type). The complete chloroplast genome was 134,555 bp in length and encoded 133 genes, including 83 protein-coding genes, 42 tRNA genes and 8 rRNA genes. Phylogenetic analysis revealed that the indica-japonica differentiation of weedy rice was closely related to cultivated rice, and *Oryza sativa* f. *spontanea* (*japonica* type) was genetically more closely clustered with cultivated rice *O. sativa* (*japonica* type) than *O. nivara* or other wild rice.

Weedy rice (*Oryza sativa* f. *spontanea* Roshev. 1931) is a noxious weed, which has threatened paddy fields worldwide and has brought enormous challenges to the sustainable crop production. It is taxonomically classified as the same species as cultivated rice (*Oryza sativa*). Like cultivated rice, weedy rice also has a significant genetic structure of *indica-japonica* differentiation (Ishikawa et al. 2005; He et al. 2017). Although the origins of weedy rice have been controversial, de-domestication from cultivated rice is considered to be a primary mechanism for the origin of weedy rice globally (Qiu et al. 2020). In addition, previous studies showed that the *indica*-*japonica* differentiation of de-domesticated weedy rice is closely associated with the *indica*-*japonica* differentiation of cultivated rice in genetic structure (Han, Ma, Cui, Wang, et al. 2020; Zhu et al. 2021). With outstanding drought tolerance, cold tolerance, salt tolerance, and water use efficiency, weedy rice has strong adaptability to barren living environment and has the potential to provide rich resources for rice genetic improvement (Sun et al. 2019; Han, Ma, Cui, Geng, et al. 2020; Li et al. 2021). Chloroplast genomes provide valuable information to species identification and evolutionary and phylogenetic studies. No complete chloroplast genome of *japonica* type weedy rice has been available. Therefore, we sequenced and assembled the complete chloroplast genome of *Oryza sativa* f. *spontanea* (*japonica* type) to explore the origin and evolution of weedy rice at the chloroplast genome level.

Phenotypically, compared to cultivated rice, weedy rice has more tillers and a shattering phenotype and the pericarp of grains is red (Supplementary Figure 1). The seeds of plants were harvested in Heilongjiang Province, China (47°43′29″N,128°45′45ʺE), planted in the agricultural experimental field of Zhejiang University, Hangzhou City, Zhejiang Province (30°18′16.9ʺN,120°4′34.68ʺE), dried and a specimen was deposited in the Herbarium of Zhejiang University under the voucher number HZU60244005 (contact person: Yiyu Hu; email: hyy_huyiyu@163.com). The whole-genome sequencing data was generated through our previous study (SRA number SRS6133688; Qiu et al. 2020).

**Figure 1.**
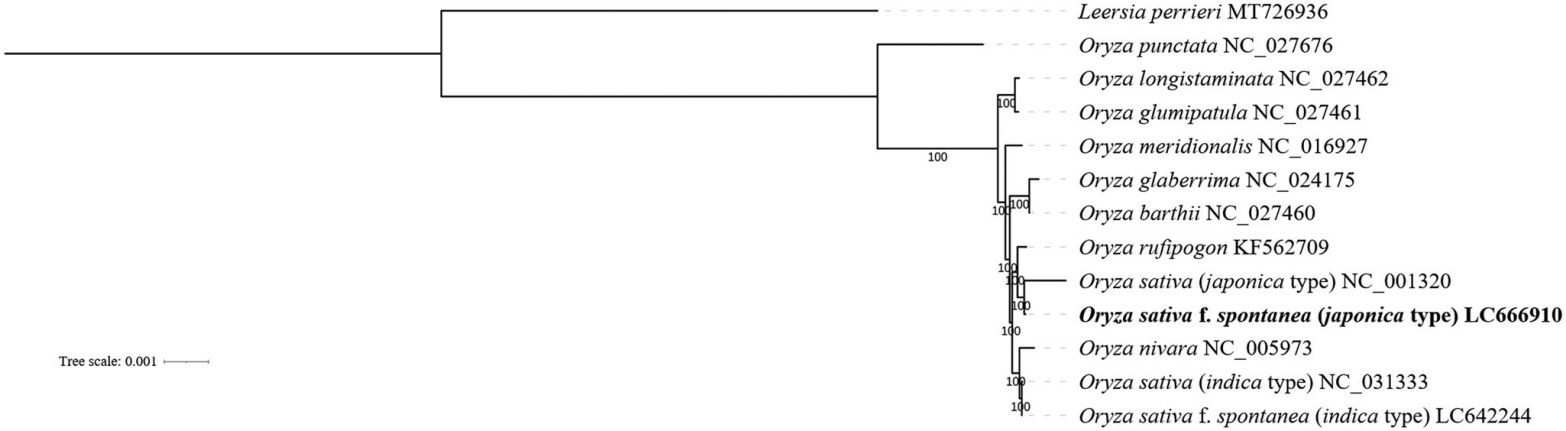
Maximum-likelihood phylogenetic tree of 13 species (varieties) based on complete chloroplast genomes (Oryza punctate, NC_027676; *Oryza longistaminata*, NC_027462; *Oryza glumipatula*, NC_027461; *Oryza meridionalis*, NC_016927; *Oryza glaberrima*, NC_024175; *Oryza barthii*, NC_027460; *Oryza nivara*, NC_005973; *Oryza sativa*(*indica* type), NC_031333; *Oryza sativa f. spontanea*(*indica* type), LC642244; *Oryza rufipogon*, KF562709; *Oryza sativa*, NC_001320; *Oryza sativa f. spontanea* (*japonica* type), LC666910 with *Leersia perrieri* as an outgroup (MT726936). Bootstrap support value from 1000 replicates is shown on each node.

First, quality control of raw sequence reads was performed using NGSQCToolkit v2.3 (Patel and Jain 2012). Then, we *de novo* assembled the clean data with NOVOPlasty v3.7.2 (Dierckxsens et al. 2017), which use complete chloroplast genome of *Oryza sativa* (GenBank accession number NC_031333) as a reference. Besides, we compared the assembled genome to reference genome (*Oryza sativa* f. *spontanea indica* type, LC642244) to detect variants by Snippy (Seemann 2015). After that, the assembled genome annotation was carried out by the GeSeq online (Tillich et al. 2017). Finally, the assembled genome sequences and annotation have been submitted to DDBJ with an accession number LC666910. The complete chloroplast genome of *Oryza sativa* f. *spontanea* (*japonica* type) was 134,555 bp in length and encoded 133 genes, including 83 protein-coding genes, 42 tRNA genes and 8 rRNA genes. The cp genome comprised four stable structures, namely a large single-copy (LSC) region of 80,604 bp, a small single-copy (SSC) region of 12,347 bp and two separated inverted repeat (IR) regions of 20,802 bp each. The GC contents in the LSC, SSC and IR regions are 37.1, 33.4, and 44.3%, respectively. 20 genes contained a single intron, and only one gene (*ycf3*) contained two introns. It duplicated 21 genes in the IR regions. Moreover, compared with *indica* type weedy rice, the chloroplast genome of *japonica* type weedy rice has 58 snps and 23 indels, and has one more *atpH* gene in the LSC region. AtpH is a chilling-repressed gene in rice, which also reveals the difference in environmental adaptability of *indica* and *japonica* type weedy rice (Mao et al. 2003).

To illustrate more clearly its evolutionary position within the *Oryza* species, we used complete chloroplast genome sequences of 11 *Oryza* species plus *Leersia perrieri* as an outgroup to construct a phylogenetic tree. Multiple sequence alignment was performed by MAFFT v7.475 (Katoh et al. 2002) with the parameters ‘–auto –reorder –phylipout.’ The alignment results were calculated using the maximum likelihood algorithm and the phylogenetic tree was constructed by IQ-tree v1.6.12 with parameters configured as ‘-m MFP -bb 1000 -bnni’ (Nguyen et al. 2015). Finally, iTOL was used to modify the constructed tree (Letunic and Bork 2019). The phylogenetic results first showed that *Oryza sativa f. spontanea* (*japonica* type) was closely clustered genetically with cultivated rice *O. sativa* (*japonica* type) rather than *O. nivara* or other wild rice (Figure 1), which again supported the previous result that *O. sativa* f. *spontanea* originated from cultivated rice and the indica-japonica differentiation of weedy rice was closely associated with cultivated rice (Qiu et al. 2017; Dong et al. 2021).

## Supplementary Material

Supplemental Material

## Data Availability

The genome sequence data that support the findings of this study are openly available in GenBank of NCBI (https://www.ncbi.nlm.nih.gov/) under the accession number LC666910, and the associated BioProject, SRA, and Bio-Sample numbers are PRJNA606132, SRS6133688, and SAMN14085922 respectively.
