# Supplementary figures and images for: The complete chloroplast genome of japonica type weedy rice (Oryza sativa f. spontanea)
Source: Mitochondrial DNA B Resour. 2022 Aug 1;7(8):1418–20. doi: 10.1080/23802359.2022.2106160 (PMC9347469; doi:10.1080/23802359.2022.2106160)

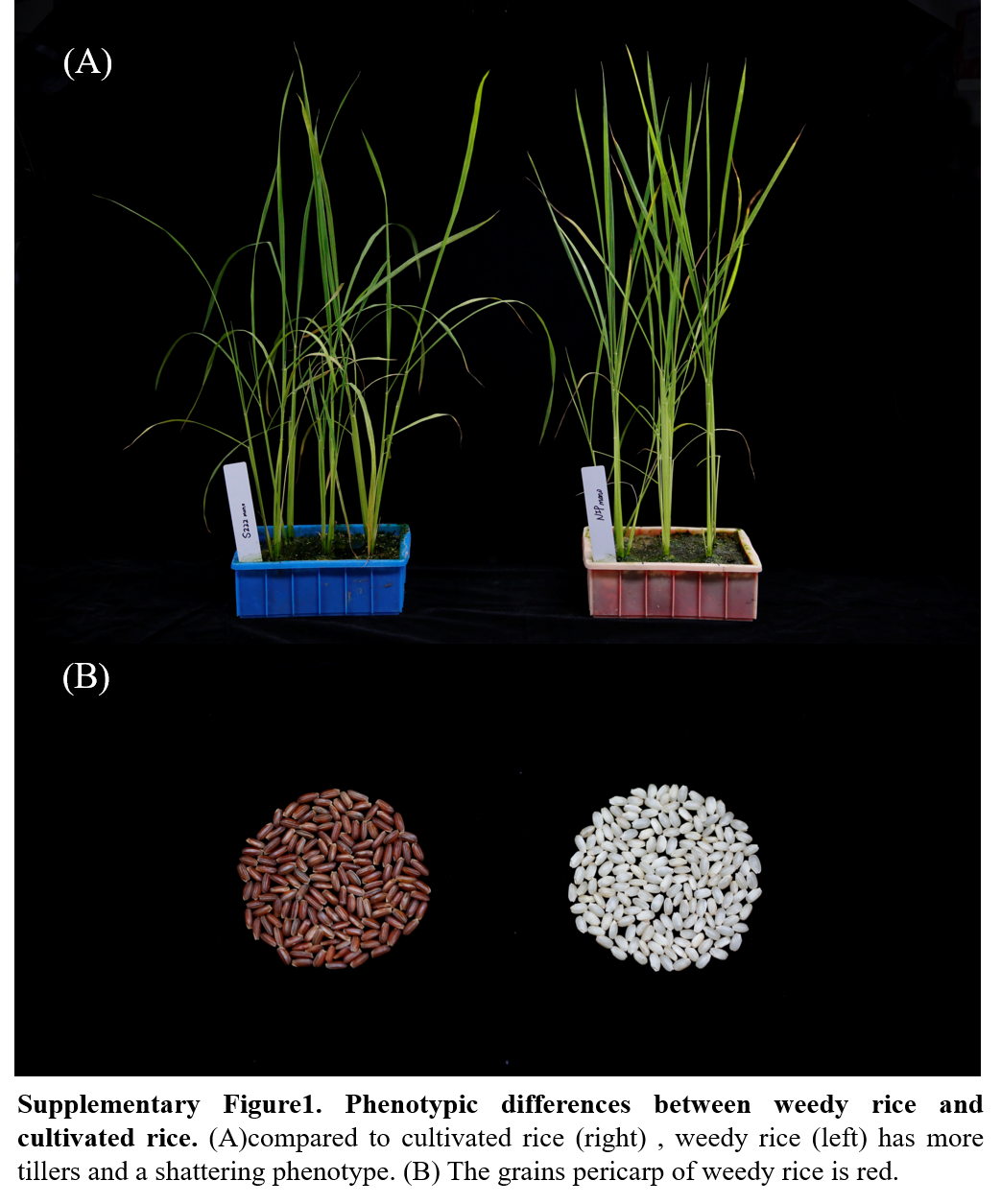

Supplement: Supplemental Material [file TMDN_A_2106160_SM5338.png]
